# Hyperosmolarity in children with hyperammonemia: a risk of brain herniation at the start of renal replacement therapy

**DOI:** 10.3389/fped.2024.1431008

**Published:** 2024-07-08

**Authors:** Yousra Maghmoul, Arnaud Wiedemann, Lucile Barcat, Fabienne Parente, Pierre Allard, Fernando Alvarez, Philippe Jouvet

**Affiliations:** ^1^ Pediatric Intensive Care Unit, Department of Pediatrics CHU Sainte-Justine, Université de Montréal, Montreal, QC, Canada; ^2^ Faculty of Medicine of Nancy, University of Lorraine, INSERM UMR_S 1256, Nutrition, Genetics, and Environmental Risk Exposure (NGERE), Nancy, France; ^3^ Biochemical and Molecular Medicine Department CHU Sainte-Justine, Université de Montréal, Montreal, QC, Canada; ^4^ Department of Pediatrics CHU Sainte-Justine, Université de Montréal, Montreal, QC, Canada; ^5^ Hepato-gastro-enterology and Nutrition Unit, Department of Pediatrics CHU Sainte-Justine, Université de Montréal, Montreal, QC, Canada

**Keywords:** hyperosmolarity, hyperammonemia, liver failure, children, brain herniation

## Abstract

**Purpose:**

Renal replacement therapy (RRT) is used in hyperammonemia to reduce the concentration of ammonia in the blood. In the case of plasma hyperosmolarity, RRT can also rapidly decrease plasma osmolarity, which may increase cerebral edema in these patients and favor the occurrence of brain herniation.

**Methods:**

We conducted a retrospective clinical study in a tertiary care university-affiliated hospital. All patients admitted in a Pediatric Intensive Care Unit (PICU), less than 18 years old with ammonemia >150 µmol/L and who underwent RRT between January 2015 and June 2023 were included. We collected data on plasma osmolarity levels, osmolar gap and blood ammonia levels before and during RRT.

**Results:**

Eleven patients were included (10 with acute liver failure and 1 with a urea cycle disorders). Their mean age was 36.2 months. Before RRT, the median highest measured osmolarity was 320 (305–324) mOsm/L, whereas the median calculated osmolarity was 303 (293–314) mOsm/L, corresponding to an osmolar gap of 14 mOsm/L. Ammonia blood level over 400 µmol/L are significantly associated with higher plasma osmolarity (*P*-Value <0.001). In one case, a patient had a brain herniation episode after a quick osmolar drop. This episode was reversed by the administration of hyperosmolar agents and the temporary suspension of RRT.

**Conclusion:**

This study highlights the hyperosmolarity and high osmolar gap that occur in children with hyperammonemia. A careful monitoring and control of plasma osmolarity evolution may alert clinician on the risk of occurrence of neurological complication such as brain herniation.

## Introduction

Ammonia (NH_3_) is a metabolic by-product resulting from the deamination of amino acids, a process catalyzed by enzymes that yield organic acids essential for synthesizing neurotransmitters and hormones. However, high concentrations of ammonia can be toxic (1). The liver converts ammonia into urea via the urea cycle (2), which is the main component of urine. Hyperammonemia is defined as an elevated blood ammonia concentration exceeding 80 µmol/L in neonates and above 50 µmol/L in infants, adolescents, and adults (3). This condition may manifest in cases of acute liver failure (ALF) as well as chronic liver failure (CLF) (4). Inherited metabolic diseases are caused by genetic defects affecting enzymes involved in human metabolism. Hyperammonemia occurs in cases of enzyme deficiencies within the urea cycle (3), as well as in organic acidurias and defects of mitochondrial fatty acid oxidation (5) due to secondary deficiencies in urea cycle metabolism (6).

Ammonia exhibits specific neurotoxic effects, manifesting with symptoms ranging from coma to seizures, and in more severe cases, can lead to death (1). This neurotoxicity is partially explained by the metabolism of glutamine by astrocytes, which result in cerebral edema (7). Treatment modalities primarily focus on reducing protein intake to limit nitrogen intake and utilizing ammonia scavengers to facilitating the elimination of excess ammonia (8, 9). Additionally, interventions targeting the intestinal microbiota, achieved through the administration of antibiotics or lactulose (10), also play a role. In cases of severe encephalopathy, or when the blood ammonia level exceeds 150 µmol/L, or if hyperammonemia proves refractory to medication, prompt initiation of renal replacement therapy (RRT) is strongly recommended to efficiently clear excessive ammonia (11).

All continuous RRT therapeutic modalities are safe and efficacious methods used for inborn errors of metabolism for more than thirty years (11–13). These treatments yield acceptable biochemical levels of ammonia in a couple of hours. Nonetheless, a notable mortality rate is still observed, often correlated with a higher concentration of ammonia in the blood (3). Hyperammonemia cases have been associated with an elevation in plasma osmolarity, resulting in an osmolar gap in afflicted patients (14, 15). But ammonia is not the only contributor to elevated osmolarity, unrecognized osmotically active molecules that accumulate in liver failure may contribute to this osmolar gap (15). Significant fluctuations in cerebral edema are documented in this population, accompanied by an increased vulnerability to neurologic complications such as brain herniation (16).

Dialysis disequilibrium syndrome (DDS), akin to that observed in patients with renal failure, may also ensue in the context of hyperammonemia. The acute reduction of blood urea through dialysis engenders osmotic shifts and consequent cerebral edema (17). Similar observations may occur in case of hyperammonemia. Thus, this study aims to document the osmolarity level at admission in children with hyperammonemia, and the evolution of plasma osmolarity during RRT.

## Material and methods

### Population

We performed a retrospective longitudinal study in our 32-bed Pediatric Intensive Care Unit (PICU) of a tertiary care university-affiliated hospital in Montréal, Québec, Canada. The unit is a medical-surgical PICU that admits all critically ill children including cardiac surgery and organ transplantation patients. We included all patients under 18 years old who underwent Renal Replacement Therapy (RRT) with an ammonemia blood level exceeding 150 µmol/L between January 2015 and June 2023, and for whom a plasma osmolarity measurement were available. We chose 150 µmol/L as the threshold for hyperammonemia based on recommendations for initiating RRT in cases with neurological manifestations (11). Blood samples for ammonia level evaluation were collected in heparin tubes and immediately placed on ice. Ammonia concentrations were measured using a Beckman Coulter LX-20 automat analyzer with spectrophotometric enzymatic method and Beckman reagents (18). Exclusion criteria included brain death and decision to withdraw or withhold therapy at PICU admission.

This study was approved by the research ethics board of CHU Sainte-Justine, protocol number 2024-6483.

### Data sources and study variables

Retrospective identification of patients was done by cross-referencing the laboratory database with the PICU database. Time zero for inclusion was defined as the time of initiation of RRT. Data were extracted from medical charts and collected with a standardized form after codification.

Data collected included sex, age, weight, patients’ underlying condition and etiology of hyperammonemia, neurologic manifestations, biological characteristics at admission, and hyperammonemia treatments (medication and/or extracorporeal removal therapies). Serum ammonia, osmolarity, and blood osmolar gap levels were noted at the time of initiation of RRT, at 3 and 6 h before RRT initiation and at 3, 6, 9, 12, 15, 18, 21, 24, and 27 h after (when data was available). When biological data were available, we calculated osmolarity according to the following formula: [2 × (Na^+^)] + [glucose] + [urea]). The osmolar gap was calculated as the difference between measured osmolarity and calculated one.

### Statistical analysis

Ammonia blood level, blood osmolarity and osmolar gap evolution were recorded for each patient. Biological data ad admission and before CRRT initiation are quoted as median [interquartile range (IQR)] and *n* values as percentages. Evolution of blood osmolarity according to time is presented as mean (+/-SD). Comparisons between patients with ammonemia blood level over 400 µmol/L and those who have ammonemia blood level under 400 µmol/L were conducted using Wilcoxon tests for continuous variables. A Spearman test was to look for a link between plasma ammonia concentration and plasma osmolarity. A *P*-Value below 0.05 was considered as statistically significant. Analyses were performed using R, version 4.3.1 (2023-06-16) (R Foundation for Statistical Computing, Vienna, Austria).

## Results

Eleven patients were included in this study. 64% are female, with a median age of 36.2 months. Their clinical and biochemical characteristics are presented in Table 1. Most patients (91%) had hyperammonemia caused by an acute liver failure (ALF), resulting in increased INR and liver enzymes (Table 2).

**Table 1:** Clinical and biochemical characteristics at PICU admission (*n* = 11).

Age (month), median (IQR)	36.2 (14.7; 132.6)
Sex (F/M), *n* (%)	7 (64)/4 (36)
Weight (kg), median (IQR)	15.0 (9.7; 44.5)
Etiology (*n*)	
Acute liver failure	10
Acetaminophen intoxication	3
Alpha-1 antitrypsin deficiency	1
Autoimmune	2
Post-liver transplant	1
Infectious (post-viral, hepatitis)	2
Autoimmune hepatitis	1
Urea cycle defect	1
Ornithine transcarbamylase deficiency	1
Biochemical parameters at PICU admission, median (IQR)	
INR	6.6 (5.0; 7.7)
AST (UI/L)	5,255 (3,199; 7,150)
ALT (UI/L)	5,264 (2,572; 7,073.5)
Glucose (mmol/L)	5.9 (3.6; 5.9)
BUN (mmol/L)	4.6 (2.1; 4.8)

INR, international normalized ratio; AST, aspartate transaminase; ALT, alanine transaminase; BUN, blood urea nitrogen. Age and weight at PICU admission. INR, AST, ALT: highest value before RRT initiation, or first value after RRT initiation when no value was available before RRT. Glucose and BUN: lowest value before RRT initiation.

**Table 2:** Individually clinical and biological (measured and calculated osmolarity) characteristics before CKKT initiation.

Patient number	Age (years)	Gender	Maximal NH_3_ blood level (µmol/L)	Pathology	INR	AST (UI/L)	ALT (UI/L)	Natremia before CRRT^a^ (mmol/L)	Glycemia before CRRT^a^ (mmol/L)	BUN before CRRT^a^ (mmol/L)	Calculated osmolarity^†^ (mOsm/L)	Measured osmolarity^a^ (mOsm/L)	Osmolar gap (mOsm/L)	Measured osmolarity^a^ (mOsm/L) at H3 after CRRT initiation
1	11.1	F	540	ALF	10.6	3,980	6,805	139	19.1	2.9	300	326	26	289
2	3.0	M	254	ALF	5.2	16,305	7,160	143	28.6	3.2	316	320	4	311
3	15.3	F	424	ALF	11.2	6,057	7,428	148	24.6	2.9	326	337	11	327
4	0.8	M	264	ALF	5.9	2,418	1,602	141	6.2	3.8	307	311	4	314
5	0.0	M	753	IEM	1.5	117	25	153	8.3	2.3	317	322	5	289
6	1.5	M	228	ALF	4.5	8,243	6,987	150	8.7	4.5	313	316	3	303^b^
7	0.9	F	210	ALF	7.8	5,255	3,506	139	6.9	0.5	285	299	14	NA
8	14.2	F	708	ALF	6,6	10,538	8,788	145	5.6	6.2	302	322	20	305
9	4.5	F	258	ALF	4.9	4,705	4,745	131	6.3	9.9	278	289	11	295
10	15.8	F	869	ALF	7.2	4,802	5,264	140	19.1	3.5	303	326	23	NA
11	1.3	F	155	ALF	7.6	701	1,638	137	7.2	0.5	282	285	3	NA

INR, international normalized ratio; AST, aspartate transaminase; ALT, alanine transaminase; BUN, blood urea nitrogen; CRRT, continuous renal replacement therapy.

^a^ Biological values are the highest before RRT initiation.

^b^ Received preventive osmotherapy at CRRT initiation.

^†^For osmolarity calculation we use the following formula: osmolarity = [2 × (Na + )] + [glucose] + [urea]).

Ammonia blood level, measured plasma osmolarity, calculated plasma osmolarity, and plasma osmolar gap before the start of RRT were increased (Table 2). Median highest measured osmolarity within 6 h before CRRT initiation was 320 (305–324) mOsm/L whereas the median calculated osmolarity was 303 (293–314) mOsm/L. The four patients with ammonia blood level above 400 µmol/L had significant higher plasma osmolarity than those under 400 µmol/L: 326 mOsm/L vs. 305 mOsm/L (*P*-Value <0.001). The spearman correlation between ammonia blood level et plasma osmolarity is 0.77 (*P*-Value = 0.006). Patients with ALF also had a significant greater osmolar gap than the others: 21.5 mOsm/L vs. 4 mOsm/L (*P*-Value = 0.031).

**Figure 1:**
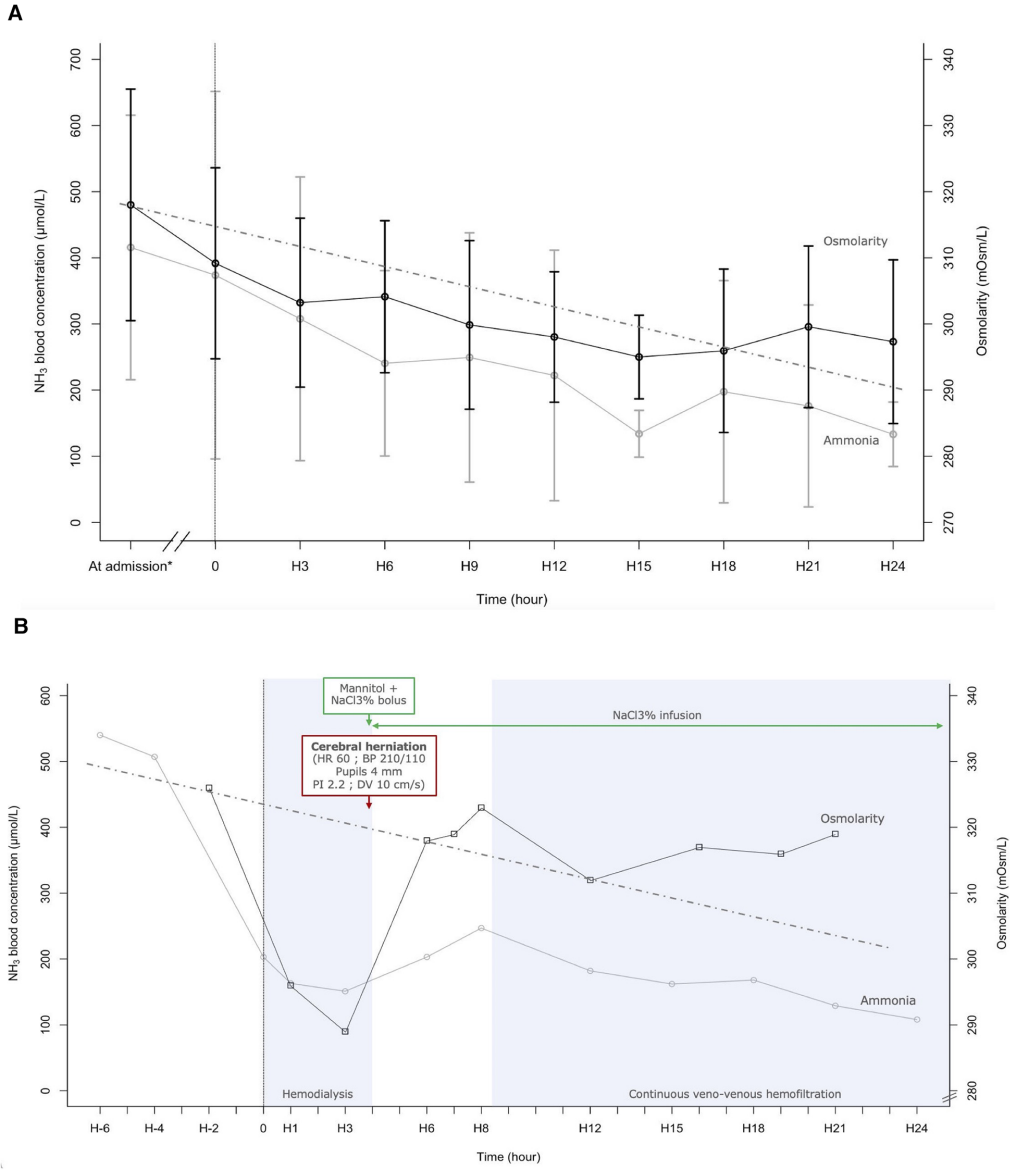
(**A**) Ammoniac blood concentration and plasma osmolarity during the 24 first hours after renal replacement therapy (RRT) initiation. Each point at “H” is an average of all measurements done 1.5 h before and after. The value at admission is an average of all values measured between 7.5 h and 1.5 h before RRT initiation. The dotted line indicates a reduction of 1 mOsm/L per hour (41). (**B**) Evolution of ammonia blood concentration and plasma osmolarity according to time and therapy, in a case of fulminant hepatitis (Patient 1). NaCl3%: 3% saline; HR, heart rate; BP, blood pressure; PI, pulsatility index; DV, diastolic velocity. This 11-year-old girl presented with fulminant hepatitis. Four hours after intermittent hemodialysis initiation (H4), she presented cushing reflex, bilateral mydriasis, and elevated intracranial pressure on transcranial doppler. cerebral herniation was reversed with cessation of hemodialysis, a bolus of mannitol and 3% saline, and with continuous infusion of 3% saline. Continuous veno-venous hemofiltration began at H8. Outcomes were good, with a complete recovery.

Plasma osmolarity and ammonia concentration within the first 24 h following RRT initiation are shown in Figure 1A. As expected, ammonia concentration decreases as the RRT progresses.

Plasma osmolarity also decreases, with the highest reduction during the first 3 h of RRT, dropping by 1.98 mOsm/L per hour. Mean osmolarity was 309 ± 14 mOsm/L at the beginning of RRT (H0), 303 ± 13 mOsm/L after 3 h of RRT (H3), and 301 ± 11 mOsm/L after 24 h of RRT (H24).

One of the patients in this study was an 11-year-old girl with an ammonia concentration at admission of 540 µmol/L (Figure 1B). Evaluation of plasma amino acid chromatography observed an nonspecific increase of amino acids concentration equivalent to 8 and 10 mOsm/L. Lactulose, intravenous sodium benzoate and N-acetylcysteine were begun. Because of an altered state of consciousness and life-threatening hyperammonemia, intermittent hemodialysis (IHD) was initiated. 2 h before starting IHD, plasma osmolarity was 326 mOsm/L. Once IHD started, the osmolarity dropped to 296 mOsm/L after 1.2 h and to 289 mOsm/L after 2.8 h. On average, there was a decrease of 8 mOsm/L per hour in the 3 first hours of IHD. After 4 h of IHD, the patient experienced the onset of cerebral herniation. The patient had bradycardia, bilaterally dilated pupils, a hypertensive episode, and a drop in cerebral blood flow seen on transcranial Doppler (Pulsatility index in the middle cerebral artery 2.2 and diastolic flow velocity at 10 cm.s^−1^ (see Supplementary Figure S1). Two boluses of 3 ml/kg of 3% saline and one bolus of 1 g/kg of mannitol were administered, and IHD was stopped. Hemodynamic parameters and pupillary diameter returned to normal after a few minutes. A continuous infusion of 3% saline was started to maintain plasma osmolarity above 300 mOsm/L. Because of persistent severe hepatic encephalopathy, continuous veno-venous hemofiltration was initiated 4 h after IHD cessation.

10 patients discharge from PICU alive after a median length of hospitalisation of 9 days (8–10.5). 1 patient required liver transplantation (Patient 6). At hospital discharge, Patient 1 had a full liver and neurological function recovery.

## Discussion

In our cohort of children with hyperammonemia, predominantly due to ALF, we observed a notable elevation in plasma osmolarity. Similar findings have been documented in critical care within the adult population affected with acute or acute-on-chronic liver failure and severe hepatic encephalopathy (14). RRT was successfully used in cases of PALF (19, 20), allowing children to be stabilized before a liver transplant or recovery. Inability to reduce ammonia by 48 h is associated with poor prognosis and death (20), which is particularly in favor of early treatment in these patients. In our study, a high level of ammonia in the blood is associated with an increase in osmolarity, partly due to many unmeasured amino acids. Consequently, rapid correction of hyperosmolarity within a few hours can have neurological consequences, as was the case for patient 1. Clinicians should be vigilant about this aspect to proactively avoid such complications.

Hepatic encephalopathy (HE) represents a significant complication not only in cases of ALF, but also in chronic liver failure (CLF) and acute-on-chronic liver failure (ACLF) (4). Pathophysiology of HE may diverge according to the underlying etiology of ALF (4). HE is a neuropsychiatric syndrome stemming from liver dysfunction, and it can manifest a wide spectrum of symptoms ranging from subtle neurologic impairment to coma and, ultimately, death because of brain herniation (21). Cerebral edema is one of the main mechanisms leading to HE. This phenomenon has been documented in both ALF and CLF (14, 22–24), and in rare congenital disorders associated with hyperammonemia (25). In case of liver failure, ammonia is not metabolized and is partially detoxified into glutamine. In the brain glutamine is osmotically active and is thought to be responsible for astrocyte swelling and cytotoxic edema seen in PALF (26, 27). Other amino acids increase are observed in case of liver failure (aromatic amino acids, sulfurated amino acids and dicarboxylic amino acids) and may have an osmolar activity (28). Ammonia increase and related amino acids disturbances, could lead to the cerebral herniation (29).

Acute hyperammonemia is a medical emergency which requires early treatment to maximize the patient's prognosis and to prevent irreversible brain injury. Typically, treatment is based on a multifaceted approach combining drugs and nutritional support in accordance with specific guidelines (3, 11, 30). Extracorporeal removal therapies, especially continuous RRT, are recommended in case of rapidly deteriorating neurological condition (coma or cerebral edema) accompanied by blood ammonia level exceeding 150 μmol/L, or in cases of severe encephalopathy, or in cases of high blood ammonia levels surpassing 400 μmol/L refractory to medical interventions (11).

Neurological manifestations are usually linked with hyperammonemia, exhibiting a dose-dependent relationship. Initially, patients may present with hypotony and lethargy, followed by seizures and coma. Pathology specimens have revealed the presence of cerebral edema with blooming astrocytes (7). Hyperammonemia leads to alterations in brain structure and its homeostasis (31), although the precise pathophysiological mechanisms remain not fully elucidated (32). Under normal physiologic conditions, astrocytes transform ammonia into glutamine by the glutamine synthetase, and release glutamine by the sodium-coupled neutral amino acid transporter (SNAT5) into the extracellular space. Accumulation of glutamine into brain leads to many pathological pathway, such as apoptosis of astrocyte, with a release of inflammatory cytokines (33), inhibit of alpha-ketoglutarate dehydrogenase, and so dysfunction of the Krebs cycle (34), decreased expression of glutamate receptors and activation of NMDA receptors by glutamate associated with calcium release (35). Hyperammonemia therefore causes cerebral suffering through different mechanisms, making neurons more sensitive to external attacks. In our cohort, we observed a significant increase in blood osmolarity co-occurring with hyperammonemia. Too rapid restoration of these imbalances, leading to a reduction in serum osmolarity, could exacerbate cerebral edema (14) like for example *in situ*ations of acute brain injury (36).

Dialysis disequilibrium syndrome (DDS) is a more common clinical situation in critical care. It is the clinical entity in which the effect of acute osmolarity change has been most studied. It manifests as a neurological complication, typically observed in patients undergoing their first hemodialysis session, resulting from a swift reduction in serum blood urea level (37). This leads to the establishment of an osmolar gradient between the brain and serum (38). Given the limited permeability of the blood-brain barrier to small molecules, water follows this osmolar gradient into the brain (36). These molecular alterations, in the presence of an osmolar gradient, facilitate the formation of cerebral edema (14). We could draw a parallel between this situation and cerebral edema due to hyperammonemia and the increase in other amino acids including glutamine.

To prevent the risk of DDS, it is widely recommended to slowly decrease the blood urea concentration. A goal of reducing urea concentration by 40% over a 2-hours span for the initial treatment is reasonable (39). Special attention should be given to children who are more susceptible to DDS (38). In cases of children with hypernatremia children, increase of cerebral edema is observed when osmolarity is reduced by 2 mOsm/L/h, and not observed when reduced by 1 mOsm/L/h (40). Current recommendations advocate for correcting chronic hypernatremia at a maximum rate of 0.5 mmol/L per hour (41), equivalent to reduction in plasma osmolarity by a maximum of 1 mOsm/L/h (40). Similarly, in hyperammonia, an osmolarity decrease of 1 mOsm/L/h could limit cerebral aggression. This rate of decrease was partially observed in our case series (Figure 1). This was not the case for patient 1, with a decrease of 37 osmol/L in 5 h (7.4 mOsm/L/h).

In cases of hyperammonemia, various RRT modalities are available. However, continuous kidney replacement therapy (CKRT), specifically high-dose CVVHD, is the recommended first-line treatment whenever feasible (11). Intermittent hemodialysis (IDH) is recommended in case of rapid deterioration in neurological status. IDH and high-dose CKRT are both considered when ammonia levels exceed 1,000 µmol/L (11). The challenge lies in the fact that clinicians aim to rapidly lower ammonia concentration but, as indicated in this study, overly swift correction may lead to severe neurological effects and could increase cerebral edema. Furthermore, the choice of RRT modality, dose, and filter membrane properties will influence the loss amino of acids and proteins in patients without protein intake (42). The loss of amino acids is directly linked to plasma amino acid concentration, CVVHD effluent volume, and filtration efficiency, assessed by the ratio of filtered urea nitrogen to blood urea nitrogen (43).

Continuous techniques appear to reduce amino acids effectively, which play a role in the genesis of the osmolar gap. Conversely, IHD demonstrates great efficacy in decreasing small molecules like ammonia, which may be responsible for DDS-like effects. In these patients, it is imperative to exercise caution and limit the decrease in osmolarity to a maximum of 1 mOsm/L/h. To limit changes in plasma osmolarity resulting from CKRT and/or IHD, the consideration of a preemptive infusion of 3% NaCl may be carefully discuss in cases of ALF with early neurocritical involvement (44).

## Conclusion

Children suffering from hyperammonemia are exposed to various neurologic complications. In both cases of AFL and rare inborn metabolic diseases, an osmolar gap link to numerous amino acids and organic acids. Close monitoring of osmolarity seems useful to adapt therapeutic management if necessary and limit neurological damage. Further studies are needed to observe the impact of different CRRT modalities on ammonia clearance and osmolar gap correction, but also impact on cerebral edema.

## Data Availability

The raw data supporting the conclusions of this article will be made available by the authors, without undue reservation.

## Supplementary material

Supplementary Figure S1 Middle cerebral artery transcranial doppler of the 11-year-old girl presented with fulminant hepatitis, before the beginning of the dialysis (A - left and B - right), during the herniation episode (C - left and D - right, see Fig 1B), and after the osmotherapy and stopping the dialysis (E - left).
