# Supplementary figures and images for: Hyperosmolarity in children with hyperammonemia: a risk of brain herniation at the start of renal replacement therapy
Source: Front Pediatr. 2024 Jul 8;12:1431008. doi: 10.3389/fped.2024.1431008 (PMC11260712; doi:10.3389/fped.2024.1431008)

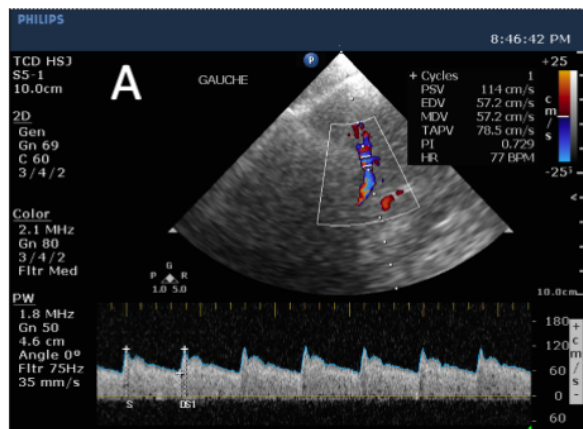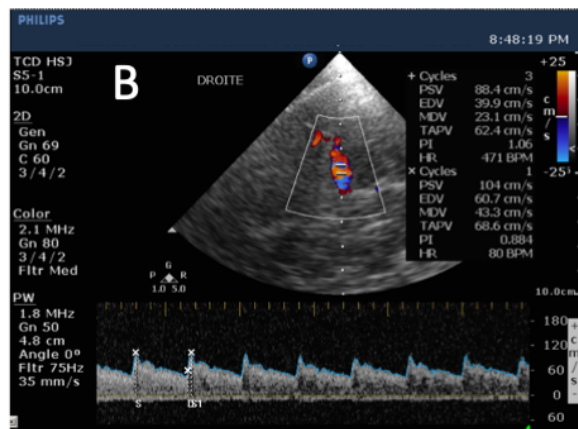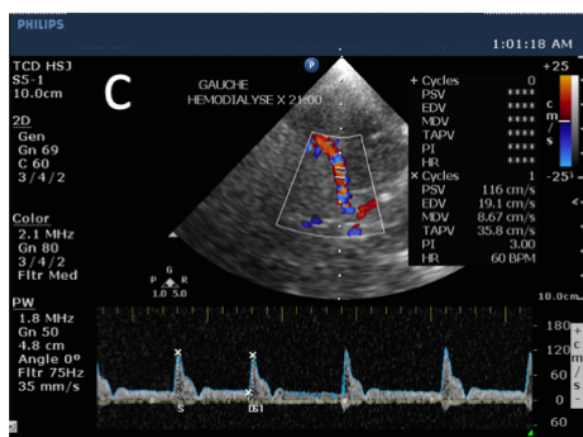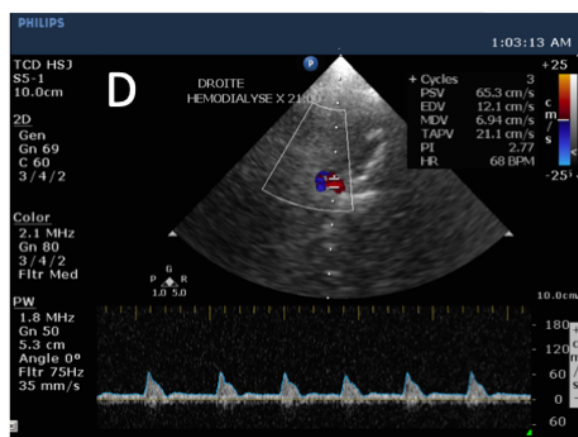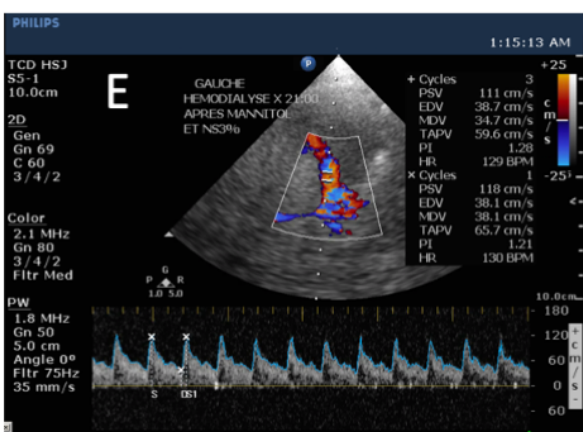

Supplement: Supplementary Figure S1 — Middle cerebral artery transcranial doppler of the 11-year-old girl presented with fulminant hepatitis, before the beginning of the dialysis (A - left and B - right), during the herniation episode (C - left and D - right, see Fig 1B), and after the osmotherapy and stopping the dialysis (E - left). [file Datasheet1.pdf]
